# Why Are Genes Encoded on the Lagging Strand of the Bacterial Genome?

**DOI:** 10.1093/gbe/evt193

**Published:** 2013-11-22

**Authors:** Xiaoshu Chen, Jianzhi Zhang

**Affiliations:** Department of Ecology and Evolutionary Biology, University of Michigan

**Keywords:** evolution, mutation-selection balance, convergence

## Abstract

Genomic DNA is used as the template for both replication and transcription, whose machineries may collide and result in mutagenesis, among other damages. Because head-on collisions are more deleterious than codirectional collisions, genes should be preferentially encoded on the leading strand to avoid head-on collisions, as is observed in most bacterial genomes examined. However, why are there still lagging strand encoded genes? Paul et al. recently proposed that these genes take advantage of the increased mutagenesis resulting from head-on collisions and are thus adaptively encoded on the lagging strand. We show that the evidence they provided is invalid and that the existence of lagging strand encoded genes is explainable by a balance between deleterious mutations that bring genes from the leading to the lagging strand and purifying selection purging such mutants. Therefore, the adaptive hypothesis is neither theoretically needed nor empirically supported.

## Lagging Strand Encoding and Head-On Collisions

**Fig. 1.—:**
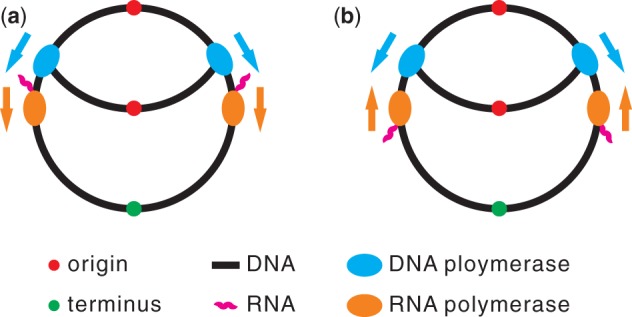
Schematic drawing of a collision between a working DNA polymerase and a working RNA polymerase in bacteria. (*a*) Codirectional collision when a gene is encoded on the leading strand. (*b*) Head-on collision when a gene is encoded on the lagging strand. Arrows show the directions of movement.

The same DNA molecule is used as the template for both replication and transcription. Consequently, DNA polymerase and RNA polymerase may collide while at work, resulting in transcriptional abortion, replication delay, and mutagenesis (Merrikh et al. 2012). When a gene is encoded on the leading strand, DNA polymerase and RNA polymerase move in the same direction (fig. 1*a*). But because in bacteria DNA polymerase proceeds 10–20 times faster than RNA polymerase (Rocha and Danchin 2003), they may collide codirectionally (fig. 1*a*). By contrast, when a gene is encoded on the lagging strand, DNA and RNA polymerases would have head-on collisions (fig. 1*b*). Because head-on collisions are more deleterious than codirectional collisions (Merrikh et al. 2012), genes should be preferentially encoded on the leading strand to avoid head-on collisions. Indeed, in most bacterial genomes surveyed, more genes are encoded on the leading than the lagging strand (Brewer 1988; Rocha and Danchin 2003; Mao et al. 2012; Merrikh et al. 2012). However, why are there still lagging strand encoded genes? In an analysis of the genome sequences of five strains of the bacterium *Bacillus subtilis*, Paul et al. (2013) concluded that these genes take advantage of the increased mutagenesis resulting from head-on collisions and are thus adaptively encoded on the lagging strand. We here show that this adaptive hypothesis is theoretically unneeded and empirically unsupported.

## Lagging Strand Encoding Can Be Explained by Mutation-Selection Balance

Let us first model the evolutionary dynamics of leading strand encoding and lagging strand encoding. Let the rate of inversion mutations that convert leading strand encoding to lagging strand encoding be *u* per gene per generation and that of the reverse mutations be *v* per gene per generation. Let *q* be the probability that a gene is encoded on the lagging strand and *s* be the selective disadvantage of lagging strand encoding of the gene relative to leading strand encoding. Under 0 < *s* ≪ 1, an equilibrium is reached when (1−*q*)*u* = *q*(*v* + *s*), or *q* = *u*/(*u* + *v* + *s*). Specifically, when *u* = *v*, *q* = 1/(2 + *s*/*u*). Thus, a balance between mutation and purifying selection can in principle explain the maintenance of lagging strand encoded genes, and the probability for a gene to be encoded on the lagging strand is determined by the ratio between the relative disadvantage of lagging strand encoding and the rate of mutation bringing the gene from the leading to the lagging strand. Interestingly, our formula predicts that *q* decreases as *s* increases, consistent with the observation that the lagging strand is underrepresented with highly expressed genes and essential genes (Brewer 1988; Rocha and Danchin 2003), which are expected to have relatively large *s*, compared with lowly expressed genes and nonessential genes, respectively.

## No Evidence for the Adaptive Hypothesis of Lagging Strand Encoding

In the five strains of *B. subtilis* analyzed by Paul et al. (2013), the number of nonsynonymous changes per nonsynonymous site (*d*_N_) is significantly greater for lagging strand encoded genes than leading strand encoded genes. For the following reasons, this difference is most likely caused by increased mutagenesis as well as reduced evolutionary constraints in lagging strand encoded genes. First, a reporter assay showed that head-on collisions induce more mutations than codirectional collisions, predicting higher mutagenesis in lagging strand encoded genes than leading strand encoded genes (Paul et al. 2013). Second, lagging strand encoded genes are enriched with lowly expressed genes and nonessential genes (Brewer 1988; Rocha and Danchin 2003), which are evolutionally less constrained than highly expressed genes and essential genes, respectively (Pal et al. 2001; Zhang and He 2005). Consistent with this interpretation, the nonsynonymous to synonymous rate ratio (*d*_N_/*d*_S_) is significantly higher for *B. subtilis* lagging strand encoded gens than leading strand encoded genes (Paul et al. 2013). Nevertheless, even for the lagging strand encoded genes, *d*_N_/*d*_S_ is generally much lower than 1 (Paul et al. 2013), suggesting that they are under purifying selection. Thus, the comparisons between the leading and lagging strand encoded genes in *d*_N_ and *d*_N_/*d*_S_ provide no evidence for positive selection on the lagging strand encoded genes.

Paul et al. (2013) argued that the elevated *d*_N_ and *d*_N_/*d*_S_ in the lagging strand encoded genes reflect positive selection because they detected a significantly higher fraction of lagging strand encoded genes than that of leading strand encoded genes to have experienced “convergent” amino acid changes. Conventionally, convergent changes refer to changes from different ancestral amino acids to the same descendant amino acid at a given position of a protein in multiple independent evolutionary lineages (Zhang and Kumar 1997; Nei and Kumar 2000). Because such changes are exceedingly unlikely to happen by chance, convergent evolution is widely viewed as an indication of adaptation (Zhang and Kumar 1997; Christin et al. 2010). However, Paul et al. (2013) used the term “convergent” to mean that a site has experienced more than one amino acid change (Paul et al. 2013), which we refer to as a multihit site. We found that none of the multihit sites they identified are convergent. A few of them have experienced parallel amino acid changes, defined by independent changes from the same ancestral amino acid to the same descendant amino acid in multiple lineages (Zhang and Kumar 1997; Nei and Kumar 2000). Although parallel evolution is also commonly believed to reflect adaptation due to its low chance probability (Zhang and Kumar 1997), the fraction of genes experiencing parallel amino acid changes is not significantly different between the leading (10/303) and lagging strands (3/34; *P* = 0.13, Fisher’s exact test). Hence, no evidence from convergent or parallel amino acid changes supports the adaptive hypothesis.

**Fig. 2.—:**
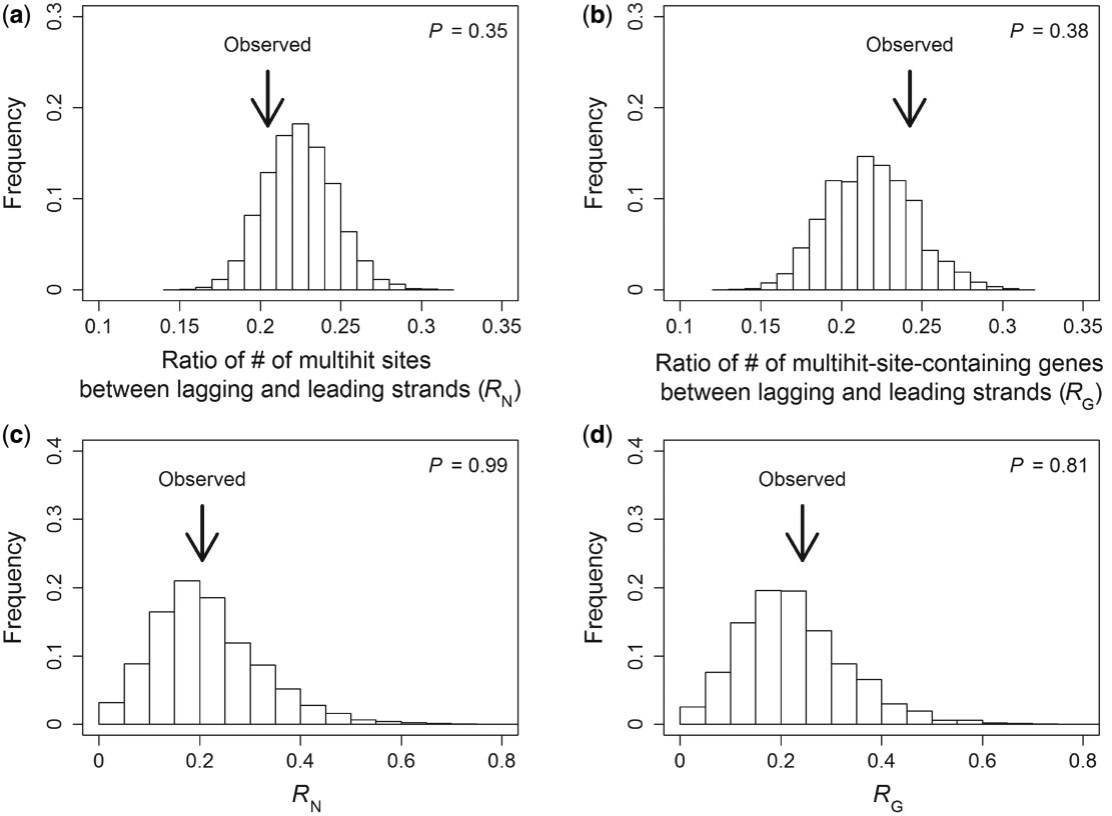
Lagging strand encoded genes do not contain more multihit sites than expected by chance. (*a*) The observed ratio (arrow) between the number of multihit sites in lagging strand encoded genes and that in leading strand encoded genes is not significantly different from the random expectation (bars). The random expectation is determined by 10,000 simulations using variable sites determined from five *B. subtilis* strains and ten additional *Bacillus* species. *P* value is the two-tail probability that a randomly generated ratio is more extreme than the observed one. (*b*) The observed ratio (arrow) between the number of multihit-site-containing genes on the lagging strand and that on the leading strand is not significantly different from the random expectation (bars). (*c*) Same as (*a*) except that all sites are assumed variable. (*d*) Same as (*b*) except that all sites are assumed variable.

Although multiple changes occurring at the same amino acid position among five strains of *B. subtilis* (i.e., multiple hits) may reflect diversifying positive selection, they may also have happened simply by chance, because the chance probability of such events is not low. In particular, compared with the leading strand, the lagging strand is expected to harbor a higher fraction of genes that contain at least one multihit site, because *d*_N_ is higher for lagging strand encoded genes. In other words, there is potentially a simpler, neutral explanation of Paul et al.’s (2013) observation about multihit sites. To examine this possibility, we randomly drew variable sites of a protein with replacement till the total number of sites drawn equals the total number of amino acid changes observed in the protein. We did this for all *B. subtilis* proteins in the data set and calculated the ratio (*R*_N_) between the total number of sites that were drawn multiple times from the lagging strand encoded genes and that from the leading strand encoded genes. We similarly calculated the ratio (*R*_G_) between the number of lagging strand encoded genes containing at least one site that was drawn multiple times and the corresponding number of leading strand encoded genes. This process was repeated 10,000 times to generate the distributions of randomly expected *R*_N_ (fig. 2*a*) and *R*_G_ (fig. 2*b*), respectively. It is clear that the *R*_N_ and *R*_G_ observed from the actual data lie in the central 90% of their respective expected distributions (fig. 2*a* and *b*). Thus, neither multihit sites nor multihit-site-containing genes are more prevalent on the lagging than the leading strand, given the different rates of amino acid changes on the two strands. In other words, Paul et al.’s (2013) observation on multihit-site-containing genes is fully expected under the neutral model. Use of different definitions of variable sites does not alter this conclusion (see Materials and Methods and fig. 2*c* and *d*).

## Conclusions

In sum, proper considerations of theory and data provide no support to the adaptive hypothesis of lagging strand encoding of bacterial genes. Rather, a mutation-selection balance can explain the evolutionary maintenance of such genes and is supported by the underrepresentation on the lagging strand of highly expressed genes and essential genes.

## Materials and Methods

The protein sequences of the five *B. subtilis* strains used by Paul et al. (2013) and ten other *Bacillus* species (*B. alcalophilus*, *B. azotoformans*, *B. cellulosilyticus*, *B. cereus*, *B. clausii*, *B. coagulans*, *B. halodurans*, *B. megaterium*, *B. pumilus*, and *B. selenitireducens*) were downloaded from EnsEMBLBacteria (release 19). One-to-one orthologs among the *B. subtilis* strains were identified by reciprocal BlastP best hits with default parameters. Following Paul et al. (2013), we focused on the subset of 334 core genes with >200 amino acids. We then used *B. subtilis* subsp. *subtilis* str. 168 proteins as queries to extract the best BlastP hits from the other ten species with protein sequence identity and length coverage both ≥70%. Multiple protein sequence alignments for the *B. subtilis* strains were generated by ClustalW with default parameters (Larkin et al. 2007). Alignment gaps were removed and the remaining sites were considered in subsequent analysis. A site is considered variable when a variant is observed in any of the 11 species (including a gap in any of the ten non-*B. subtilis* species). We also considered all scenarios when there are additional variable sites until all sites in a protein are variable, but the results are qualitatively the same (fig. 2*c* and *d*). The list of multihit sites and the amino acid changes at these sites (among the five *B. subtilis* strains) were kindly provided by Paul et al. (2013). Amino acid changes at all other sites were identified from protein sequence alignments of the *B. subtilis* strains.
